# Beyond Temporary Sobriety: The Association Between Sobriety Campaign Completion and Intention to Quit Alcohol

**DOI:** 10.3390/ijerph23070912

**Published:** 2026-07-16

**Authors:** Nittaya Srisuk, Manolee Sripaoraya Penpong, Paithoon Sonthon, Tharin Phenwan, Paul Toner, Udomsak Saengow

**Affiliations:** 1 Faculty of Nursing, Rajamangala University of Technology Thanyaburi, Klong Laung, Pathum Thani 12110, Thailand; nittaya_sr@rmutt.ac.th; 2 Faculty of Management, Suratthani Rajabhat University, Mueang Surat Thani, Surat Thani 84100, Thailand; 3 Faculty of Science and Technology, Phetchabun Rajabhat University, Mueang Phetchabun, Phetchabun 67000, Thailand; paithoon.son@pcru.ac.th; 4 Faculty of Health, University of Dundee, Dundee DD1 4HJ, UKptoner001@dundee.ac.uk (P.T.); 5 Center of Excellence in Data Science for Health Study, Walailak University, Tha Sala, Nakhon Si Thammarat 80160, Thailand; 6 School of Medicine, Walailak University, Tha Sala, Nakhon Si Thammarat 80160, Thailand

**Keywords:** alcohol, abstinence, sobriety, intention to quit, campaign, public health intervention

## Abstract

**Highlights:**

**Public health relevance—How does this work relate to a public health issue?**

- Sobriety campaigns, defined as campaigns that encourage drinkers to temporarily refrain from drinking, have been organized in several countries.
- Existing evidence suggests that sobriety campaigns can reduce population-level alcohol consumption and that individual-level reductions can persist several months beyond the campaign period.

**Public health significance—Why is this work of significance to public health?**

- This study is among the first to examine the relationship between sobriety campaign completion and intention to quit drinking, which is a longer-term behavior.
- The study demonstrates a strong association between completing the campaign and intention to quit (adjusted OR = 4.75; 95% CI, 3.17–7.34).

**Public health implications—What are the key implications or messages for practitioners, policy makers and/or researchers in public health?**

- For policymakers, sobriety campaigns should be considered as a strategy to encourage drinkers to reduce their alcohol consumption and quit drinking, complementing the WHO’s recommended interventions.
- For practitioners, integrating cessation support or follow-up resources into sobriety campaigns may support the translation of elevated quitting intention into sustained behavior change.

**Abstract:**

Sobriety campaigns (e.g., Dry January (UK), Dry July (Australia), and Thailand’s three-month sobriety campaign) have emerged as strategies to reduce alcohol use, with evidence suggesting sustained effects of such campaigns on drinking behavior for several months post-campaign. However, the relationship between campaign completion and intention to quit (a predictor of quitting behavior) remains underexplored. This study aims to examine the association between completing a sobriety campaign and intention to quit drinking. This is a pooled cross-sectional analysis of three waves of a nationally representative survey, using multivariate logistic regression. Intention to quit drinking was the dependent variable, and completion of the campaign (completely abstaining versus decrease in drinking) was the explanatory variable in the model, adjusting for demographic and drinking-related covariates. Overall, 12.6% of those completing the recent-year campaign expressed an intention to quit drinking. Completing the recent-year campaign was strongly associated with higher odds of intention to quit (adjusted OR 4.75; 95% CI, 3.17–7.34). The findings suggest that temporary sobriety campaigns may serve as an entry point for longer-term behavioral change. Policymakers should consider sobriety campaigns as a complementary strategy to reduce alcohol use alongside the WHO’s recommended interventions.

## 1. Introduction

Alcohol consumption is an important global health problem. In 2019, the use of alcohol led to 2.6 million deaths worldwide [1]. It is a risk factor for a wide range of health problems, such as lower respiratory tract infections, cancers, cirrhosis, cardiovascular diseases, and injuries [2]. Harm from alcohol extends beyond drinkers, also including harm to others (such as through diminished health, safety, and well-being) [3]. Household members, family members, and friends, as well as co-workers, are among those affected by the drinker’s behaviors [4,5]. Among adolescents, higher alcohol intake has been associated with lower academic performance and an increased risk of school dropout [6,7]. Countries have put in place various measures to control alcohol consumption and its consequences. Effective measures include restrictions on alcohol availability, alcohol advertising, sponsorship, and promotion; alcohol excise taxes and pricing policies; drink driving countermeasures; and screening, brief interventions and treatment [8].

In addition to these measures, sobriety campaigns (campaigns that encourage drinkers to refrain temporarily, for one to three months, from drinking alcohol) have been organized in several countries. Among the earliest of these campaigns, the Thai three-month sobriety campaign, which promotes three months of sobriety during Buddhist Lent (a period that typically falls between July and October), has been held annually since 2003 [9]. In Australia and New Zealand, there is a campaign called Dry July, which promotes sobriety in the month of July. It has been held since 2008 [10]. The Dry January campaign, which started in 2013, encourages one month of sobriety in January following the Christmas–New Year holidays [11]. Dry January has inspired similar campaigns in many countries, including IkPas (“No Thanks”) in the Netherlands and Tournée Minérale in Belgium [12,13].

A study using 23-year time-series alcohol sales data to evaluate the effectiveness of the Thai three-month sobriety campaign found that the campaign was associated with an average 10% reduction in alcohol consumption during the campaign period [14]. A third of Thai drinkers reportedly abstained from drinking during the sobriety campaign period [9]. In contrast, an analysis of 37,142 survey respondents showed that an increase in Dry January participation between 2015 and 2018 was not associated with a significant change in self-reported alcohol consumption [15]. Sales data are a more reliable measurement of alcohol consumption than self-reported alcohol consumption (which is generally underestimated) at the population level [16]. Hence, sobriety campaigns are potentially associated with up to a 10% reduction in population-level consumption.

Longer-term effects of sobriety campaigns have been demonstrated. Reductions in consumption were shown to persist between three to eight months after campaign completion in Thailand, the UK, and Belgium [17,18,19]. A significant increase in drink refusal self-efficacy at one and six months following Dry January was shown [17,20]. Improvements in both physical and mental well-being from baseline were reported six months after Dry January. A significantly lower Alcohol Use Disorders Identification Test-Concise (AUDIT-C) score was also observed six months after Dry January [20]. Drinkers who completely abstained throughout campaign periods had a greater reduction in alcohol consumption at the six-month follow-up [17,20].

No previous study has examined whether completing a sobriety campaign influences drinkers’ intention to quit, a well-established predictor of subsequent cessation behavior in tobacco research [21,22,23]. To address this gap, this study aimed to investigate the association between completing a sobriety campaign and intention to quit drinking using data from the Thai sobriety campaign survey. The hypothesis was that completing sobriety campaigns is associated with an increased likelihood of intention to quit drinking.

## 2. Methods

### 2.1. Study Design

The study design was an analysis of pooled cross-sectional surveys. This study analyzed pooled data from three waves (2015, 2018, and 2021) of the Buddhist Lent Abstinence Evaluation Survey. The study protocol was approved by the Human Research Ethics Committee of Walailak University, Nakhon Si Thammarat, Thailand (WU-EC-MD-3-090-65).

### 2.2. Data Source

The Buddhist Lent Abstinence Evaluation Survey is an in-person survey conducted annually by the Research Centre for Social and Business Development and funded by the Center for Alcohol Studies (CAS) to evaluate the Buddhist Lent Abstinence campaign [24], which has been listed in Thailand’s National Alcohol Strategy under the altering social norms toward alcohol and reducing drinking motivation strategy [25]. In each survey wave, data collection was conducted over a two-week period at the end of the Buddhist Lent period each year (around September–October). The target population of the survey was the general population aged 15 years old and older, which is consistent with the age range used in the Smoking and Drinking Behavior Survey—a national alcohol survey [26].

Survey respondents were selected using multistage sampling. Firstly, all provinces in Thailand were stratified into five strata, including the Bangkok metropolitan region (i.e., Bangkok and its surrounding provinces), central region, northern region, northeastern region, and southern region. Two to three provinces were randomly selected from each stratum. Districts were randomly selected from each province, followed by subdistricts. Then, sampled households were randomly selected from each sampled subdistrict. Finally, one household member who satisfied the eligibility criteria was invited to participate in the survey. The number of selected units was proportional to the size of the target population at each stage of sampling. The survey is nationally representative.

Survey items consisted of demographic characteristics, campaign-related items, and alcohol-related items. Items in the questionnaires slightly differed between each wave of the survey. Three waves of the survey in 2015, 2018, and 2021 were selected because they comprise all variables required for answering the research questions. All variables included in this analysis were consistently measured in each wave. In total, there were 14,942 respondents from three waves combined. The dataset used in this analysis was accessed with permission from CAS, which funded the survey and owns the dataset. CAS granted the research team access to the dataset for the purposes of this study.

### 2.3. Data Management

Variables included in the analysis were grouped into three categories: demographic, campaign-related, and drinking-related variables. Demographic variables included sex, age, education, religion, and monthly income. Campaign-related variables comprised completing recent-year campaign, completing former-year campaign, and exposure to campaign media. Drinking-related variables included intention to quit drinking, drinking frequency in the past 12 months (prior to the campaign), past-year episode of drunkenness, and drinking expenses per occasion.

Sex was classified into two categories (male and female). Age had five categories: 15–19, 20–30, 31–45, 46–60, and ≥61 years old. Education was classified into three levels: elementary, secondary, and bachelor’s degree or higher. Religion had two categories: Buddhism and others. Religions other than Buddhism were grouped together because more than 90% of the Thai population is Buddhist [27]. Income was classified into five levels: 0–4999 Thai baht (THB), 5000–9999 THB, 10,000–19,999 THB, 20,000–29,999 THB, and ≥30,000 THB.

The primary outcome was intention to quit drinking. The item for intention to quit drinking was “After the Buddhist Lent, do you intend to continue abstinence from drinking?”; the possible responses were resumption of drinking as usual, reducing or abstaining from drinking for a certain period, and quitting drinking. The responses were classified as ‘Yes’ for quitting drinking and ‘No’ otherwise. Intention to quit is a widely used predictor of smoking cessation in tobacco research [21,22,23]. Its association with actual quitting behavior has been well established. For instance, a follow-up study on adolescent smoking showed that intention to quit was an independent predictor of quit attempts in the next 30 days [28].

Completing a recent-year campaign was assessed by the item “From the beginning of the Buddhist Lent period until now, do you abstain from drinking alcohol?” Responses were classified as ‘Yes’ for complete abstinence from drinking and ‘No’ otherwise. Completing a former-year campaign was classified using the same approach. The Buddhist Lent campaign is a nationwide mass media campaign that encourages drinkers to voluntarily abstain from alcohol during the three-month Buddhist Lent period. Successful campaign completion was defined as complete abstinence throughout the campaign among individuals who had consumed alcohol within the 12 months preceding the start of the campaign. The same definition was used in the previous study [24]. Exposure to the campaign media variable was classified as ‘Yes’ and ‘No’. This variable was included in the analysis because previous research showed it was associated with campaign completion [24].

Three indicators of drinking patterns were classified as follows. Drinking frequency in the past 12 months was classified as weekly (≥1 time per week), monthly (1–3 times per month), and occasionally (<1 time per month). Past-year drunkenness was measured as the number of intoxication episodes in the past 12 months and classified into three levels: never, occasionally (1–2 times), and ≥3 times. Both self-reported measures are well-established correlates of alcohol-related harm [29,30]. Drinking expenses per occasion were categorized into four levels: <300 THB, 300–499 THB, 500–999 THB, and ≥1000 THB. This variable was included because anticipated financial saving from reduced alcohol consumption is a key motivator for campaign participation.

**Figure 1:**
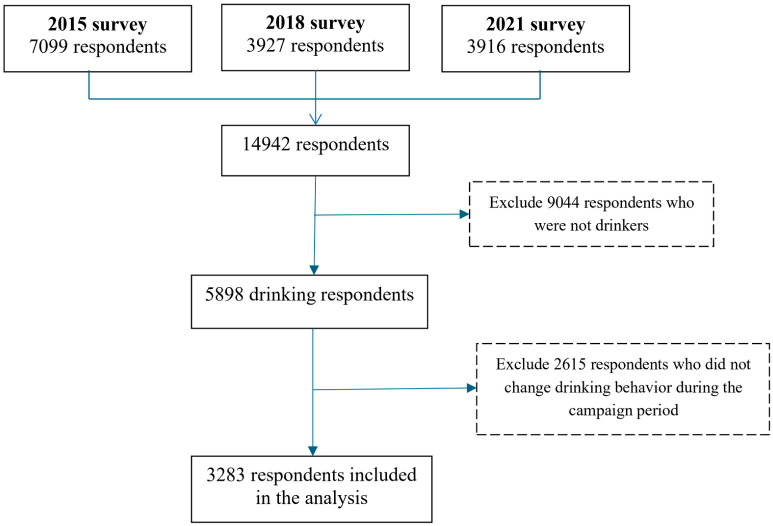
Selection of respondents for the analysis.

Given that the intention to quit drinking was relevant only to drinkers, respondents who did not drink in the past 12 months prior to the campaign were excluded from the analysis. According to the survey design, only respondents who changed their drinking behavior during the campaign period were asked the intention to quit item. Hence, respondents who continued their drinking as usual were also excluded. Finally, data from 3283 respondents (who were either drinking reducers or campaign completers) were included in the analysis (Figure 1).

### 2.4. Statistical Analysis

Respondent characteristics were summarized using percentages. The association between intention to quit drinking and each characteristic (bivariate analysis) was determined using the chi-squared test. All analysis was unweighted because the primary aim of this study was to estimate associations among variables within the pooled dataset. Combining design weights across three survey waves would require additional assumptions and lead to lower efficiency and statistical power. Furthermore, previous methodological studies demonstrated that unweighted analyses often have estimates that do not substantially differ from weighted estimates [31,32].

To determine whether completing a campaign was associated with intention to quit, multivariate logistic regression was employed. In the regression, intention to quit drinking was the dependent variable. The explanatory variables of interest were completing a recent-year campaign and completing a former-year campaign. Completing a recent-year campaign was the main explanatory variable of interest. Completing a former-year campaign was included to test whether there is a lasting effect of this variable. Therefore, two competing regression models were performed. The first model included both completing recent-year campaign and completing former-year campaign variables. The completing former-year campaign variable was removed in the second model. Other covariates (i.e., exposure to campaign media, drinking frequency in the past 12 months, past-year episode of drunkenness, drinking expenses per occasion, sex, age, education, religion, and monthly income) were similar in both models. The final model was selected based on the Akaike Information Criterion (AIC), where a model with the lowest AIC is generally considered the best balance of fit and parsimony [33,34]. The designated sensitivity analysis included all variables in the first model with the addition of the year of survey variable. Respondents with missing data on any variables included in the models were excluded from the regression analysis, which was unweighted. The magnitude of association was represented by odds ratios (ORs) and 95% confidence intervals (95% CIs). As the data in the analysis included only campaign completers and drinking reducers, the ORs of the completing campaign variables indicated the intention of campaign completers compared to that of drinking reducers. Both subgroups had already demonstrated some motivation to change their drinking behavior through participation in the campaign. R software version 4.0.3 was used to perform all analysis. The forest plot was created using ggplot2() and dplyr().

## 3. Results

Respondent characteristics of the pooled dataset are shown in Table 1. The majority were male (63.8%). The average age was 38.6 years old. Most (97.6%) were Buddhist. Three quarters of respondents had secondary education or higher. Around half spent less than 300 THB per drinking occasion. Among the respondents, 70% drank at least once a month; 22.4% had three or more episodes of drunkenness in the past 12 months. Most were exposed to the Buddhist Lent Abstinence campaign media. Overall, 6.9% intended to quit drinking.

Table 2 presents the percentages for intention to quit (unweighted) by characteristics. The following groups had percentages of quitting intention >10%: individuals who completed the recent-year campaign, those who completed the former-year campaign, occasional drinkers, those without an episode of drunkenness in the past 12 months, and respondents aged 61 years and older. The bivariate analysis indicated that intention to quit drinking was significantly associated with completing a recent-year campaign, completing a former-year campaign, drinking frequency, past-year episode of drunkenness, drinking expenses per occasion, sex, age, and monthly income.

**Figure 2:**
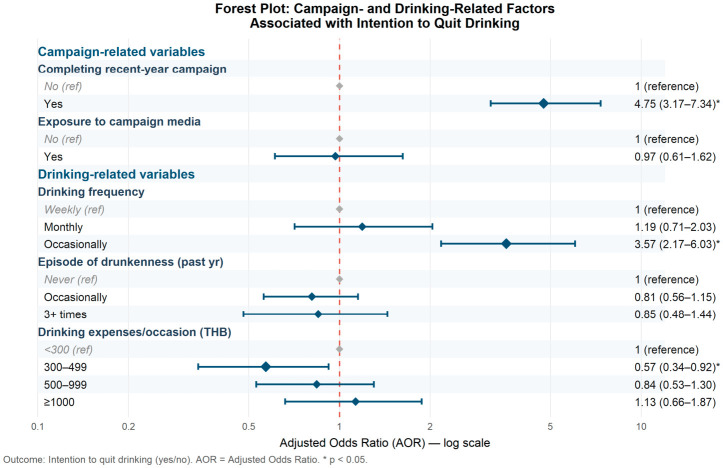
Factors associated with intention to quit drinking (multivariate logistic regression). Note: n = 2820 (463 records contained missing data); adjusted for sex, age, education, religion, and monthly income (see Table S2 for coefficients); and completing recent-year campaign, exposure to campaign media, drinking frequency, past-year episode of drunkenness, and drinking expenses per occasion (shown in Figure 2). See Table S2 for the full regression model.

The forest plot in Figure 2 presents the results of the final multivariate regression model. This model was selected as the most parsimonious model among models with very close AIC values (see Tables S2–S4). This model included all covariates except completing a former-year campaign; 463 records (14.1%) were excluded from the final model due to missing covariate data. In this model, completing a recent-year campaign was significantly associated with intention to quit drinking, with an OR of 4.75 (95% CI, 3.17–7.34). Occasional drinkers had 3.57 times the odds of intention to quit compared to those who drank weekly (95% CI, 2.17–6.03). Age and monthly income were also associated with intention to quit (see Table S2 for the full regression model). The regression model with all covariates was used as sensitivity analysis 1, and the regression analysis with all covariates plus the year variable was regarded as sensitivity analysis 2. The findings from these three models were consistent. The year variable showed no significant association with intention to quit. This demonstrates the robustness of the observed associations, although the estimated odds ratios differed slightly. The full models are provided in the Supplementary Materials (Tables S3 and S4).

## 4. Discussion

By analyzing data from 3283 drinkers across three waves of a nationally representative survey, this study found a strong association between completing the recent-year sobriety campaign and intention to quit drinking (adjusted OR 4.75; 95% CI, 3.17–7.34), with 12.6% of completers stating this intention. Occasional drinkers had significantly higher odds of intention to quit compared to those who drank weekly. These findings suggest that the benefits of sobriety campaigns may extend beyond short-term reductions in alcohol consumption to include increased intention to quit drinking, an established predictor of subsequent cessation behavior.

Existing evidence indicates that reductions in alcohol consumption in sobriety campaign participants can persist beyond the campaign periods [17,18,19]. In an evaluation of the Thai sobriety campaign at the community level, communities with campaign-related activities (in addition to the national mass media campaign) had a significantly higher rate of abstinence compared to communities without the additional activities for up to three months after the campaign ended [18]. In the UK, a six-month follow-up study of the Dry January campaign found that those who completed the campaign had greater reductions in drinking frequency, quantity, and drunk episodes than those who did not complete the campaign [17]. Similarly, the Belgian sobriety campaign showed a significant reduction in the number of drinks consumed per week at eight-month follow-up compared with pre-campaign consumption [19]. The present study extends this evidence by showing that sobriety campaign completion is associated with intention to quit drinking among a meaningful proportion of campaign participants, indicating that such campaigns may be linked to the initiation of behavioral change.

Cultural congruence may be an important condition for the participation and effectiveness of sobriety campaigns. Dry January in the UK, IkPas in the Netherlands, and Tournée Minérale in Belgium are secular campaigns framed around health benefits and self-improvement [11,12,13]. In contrast, the Thai campaign is anchored in Buddhist Lent, a religious observance during which refraining from alcohol is consistent with the fifth Buddhist precept [35]. This alignment between the campaign and prevailing cultural values may encourage participation and campaign completion among the predominantly Buddhist population in Thailand. This interpretation is consistent with evidence that religious affiliation and religiosity are among the strongest correlates of alcohol abstention [36]. Previous research has shown that messages are more persuasive when their source, content, and delivery channel are congruent with the culture of the target audience [37]. These findings suggest that sobriety campaigns should not be regarded as culturally neutral interventions. Rather, their effectiveness may depend partly on how well the campaign aligns with the cultural context of the target population. Transferring campaign models across settings may require deliberate cultural adaptation.

Age was associated with intention to quit in the adjusted model, with the highest proportion of quitting intention observed in older age groups. Aging is accompanied by declining tolerance of alcohol, accumulating health problems, and more frequent contact with health services, all of which may prompt older drinkers to consider quitting [38]. Monthly income remained associated with quitting intention after adjustment, with higher intention to quit observed among those in lower income groups. As research on alcohol quitting intention is limited, evidence from tobacco control provides a relevant comparison. Our findings are consistent with a study from Thailand and Malaysia; smokers with higher incomes were less likely to intend to quit than those with lower incomes [39]. However, studies conducted in high-income countries have consistently reported higher quit intentions and attempts among higher income groups [21,40]. One possible explanation is that alcohol expenditure represents a greater proportion of income among lower-income populations in middle-income countries such as Thailand than in high-income countries. Hence, the potential financial benefits of reducing or stopping alcohol consumption may be more prominent for lower-income drinkers, thereby strengthening their intention to quit. The 2021 wave of the surveys was collected during the COVID-19 pandemic. In Thailand, control measures included restrictions on on-premises alcohol consumption, closure of entertainment venues, and limits on social gatherings. Nationally representative surveys conducted during the 2020 restrictions found that about half of Thai drinkers abstained during the alcohol sales ban and one-third drank less than usual, while almost none reported drinking more [41]. Although the year of survey showed no significant association with the intention to quit in this analysis, the OR for 2021 suggested lower quitting intention. The unintentional reduction in alcohol consumption due to the lower availability of alcoholic beverages may lead to cravings [42,43], hence less intention to quit.

The intention to quit drinking, while not a direct measure of behavior change, is a well-established predictor of future action in health behavior research. According to the Theory of Planned Behavior, intention is the most proximal determinant of behavior and is influenced by attitudes toward the behavior, perceived social norms, and perceived behavioral control [44]. In the context of alcohol abstinence, individuals who express an intention to quit are likely to have formed favorable attitudes toward sobriety, perceive social support for quitting, and believe in their ability to maintain abstinence. This theoretical framework supports the use of intention to quit as a meaningful surrogate outcome in this study. Moreover, extensions of the Theory of Planned Behavior, such as the Health Action Process Approach, emphasize the importance of post-intentional processes like planning and self-efficacy in translating intention into sustained behavior change [45]. These models suggest that campaign-related activities—such as making public commitments about sobriety, social reinforcement, and exposure to supportive messaging—may strengthen the intention–behavior link. Although the present study focused on intention to quit, future research should incorporate longitudinal designs to assess actual quitting behavior and explore mediating factors that facilitate or hinder the transition from intention to action.

According to the findings of this study, a temporary abstinence campaign, a relatively low-cost intervention, could complement the measures recommended in the WHO SAFER technical package [8]. The finding that 12.6% of campaign completers intended to quit drinking indicates that the end of the temporary-abstinence period is a strategic window for prevention policy. Strategies that could be deployed at the end of the campaign include booster messaging encouraging sustained abstinence or reduced drinking, screening and brief interventions, and referral pathways to cessation services.

This study has several strengths. The study utilizes a large, nationally representative dataset spanning three waves of the Buddhist Lent Abstinence Evaluation Survey, enhancing the generalizability of the findings within the Thai context. The use of multistage sampling and stratification across geographic regions ensures robust population coverage and diversity in respondent characteristics. Moreover, the study focuses on a unique and culturally embedded sobriety campaign, providing valuable insights into public health interventions that integrate religious and social norms. Next, the analysis distinguishes between recent and past campaign participation, allowing for the assessment of both immediate and potential lasting associations with intention to quit drinking. Finally, the study contributes to the literature by linking campaign participation to intention to quit drinking, a relatively underexplored outcome in alcohol research, and situates this within established behavioral theories, thereby strengthening its conceptual foundation.

Nonetheless, this study has several limitations. Respondents who did not change their drinking behavior during the campaign were excluded (due to missing data on the intention-to-quit item). This may have introduced selection bias toward more motivated individuals. Consequently, the estimated ORs reflect the intention to quit among campaign completers relative to drinkers who had already reduced their alcohol consumption (both groups representing individuals with some degree of motivation to change). Therefore, the findings should be interpreted and generalized only within this population rather than to all drinkers. The cross-sectional design limits the ability to establish causal relationships between campaign participation and intention to quit drinking. It is also possible that individuals with a pre-existing motivation to quit (before the campaign started) were more likely to complete the campaign. This makes it difficult to determine whether campaign completion increased quitting intention or vice versa. A longitudinal study would allow for stronger causal inference by tracking changes in actual drinking behavior over time. Specifically, a prospective design would establish a clear temporal sequence by ensuring that campaign exposure precedes the development of quitting intentions. This approach would allow researchers to distinguish between short-term abstinence and sustained reductions in alcohol consumption.

Finally, the findings are based on the Thai cultural context, which may limit generalizability to other settings. Comparative studies across different cultural contexts or multi-country surveys could help assess the broader applicability of sobriety campaigns. Additionally, incorporating psychological constructs, such as drinking refusal self-efficacy and the perceived approval of family and friends, would allow regression models to account for unobserved heterogeneity among drinkers. This would explain a greater proportion of the variance in quitting intentions and improve the predictive performance of the models.

## 5. Conclusions

This study demonstrates a strong association between completing a sobriety campaign and intention to quit drinking, with 12.6% of recent-year completers expressing this intention (adjusted OR 4.75; 95% CI, 3.17–7.34). These findings align with and extend existing evidence by suggesting that the benefits of sobriety campaigns may extend beyond temporary reductions in alcohol consumption to include increased intention to quit drinking. For policymakers, the results support the inclusion of sobriety campaigns as a complementary strategy alongside the WHO’s recommended alcohol control measures. Furthermore, integrating cessation support into sobriety campaigns may help translate increased intention to quit into sustained behavior change. Future research should employ longitudinal designs to determine whether intention to quit leads to actual cessation, examine mediating mechanisms such as self-efficacy and social support, and conduct comparative studies across cultural contexts to assess the generalizability of these findings.

## Figures and Tables

**Table 1:** Demographic characteristics of the sample (N = 3283).

Characteristic	Total	
	n	%
Demographic variables		
Sex		
Male	2093	63.8
Female	1190	36.2
Age (years)		
15–19	189	5.8
20–30	839	25.6
31–45	1184	36.1
46–60	898	27.4
≥61	167	5.1
missing	6	0.2
Education		
Elementary	751	22.9
Secondary	1470	44.8
Bachelor and beyond	1045	31.8
missing	17	0.5
Religion		
Buddhism	3203	97.6
Others	46	1.4
missing	34	1.0
Monthly income (THB)		
<5000	367	11.2
5000–9999	985	30.0
10,000–19,999	1272	38.7
20,000–29,999	446	13.6
≥30,000	186	5.7
missing	27	0.8
Campaign-related variables		
Exposure to campaign media		
No	407	12.4
Yes	2876	87.6
Completing recent-year campaign		
No	1718	52.3
Yes	1565	47.7
Completing former-year campaign		
No	1847	56.3
Yes	1426	43.4
missing	10	0.3
Drinking-related variables		
Intention to quit drinking		
No	2984	90.9
Yes	227	6.9
missing	72	2.2
Drinking frequency in the past 12 months		
Weekly	998	30.4
Monthly	1275	38.8
Occasionally	985	30.0
missing	25	0.8
Past-year episode of drunkenness		
Never	1371	41.8
Occasionally	1167	35.5
≥3 times	736	22.4
missing	9	0.3
Drinking expenses per occasion (THB)		
<300	1470	44.8
300–499	623	19.0
500–999	502	15.3
≥1000	341	10.4
missing	347	10.6

**Table 2:** Bivariate analysis of respondent characteristics and intention to quit drinking.

Characteristic	Intention to Quit (Unweighted)		*p*-Value ^t^
	%	95% CI	
Campaign-related variables			
Exposure to campaign media			0.611
No	7.3	4.8, 10.9	
Yes	7.6	6.6, 8.7	
Completing recent-year campaign			<0.001 *
No	2.1	1.5, 2.9	
Yes	12.6	11.0, 14.3	
Completing former-year campaign			<0.001 *
No	3.3	2.5, 4.2	
Yes	12.0	10.4, 13.8	
Drinking-related variables			
Drinking frequency in the past 12 months			<0.001 *
Weekly	3.1	2.2, 4.4	
Monthly	4.4	3.4, 5.7	
Occasionally	14.1	12.1, 16.5	
Past-year episode of drunkenness			<0.001 *
Never	10.3	8.8, 12.1	
Occasionally	5.7	4.4, 7.2	
≥3 times	3.2	2.1, 4.9	
Drinking expenses per occasion (THB)			0.004 *
<300	8.0	6.7, 9.5	
300–499	3.6	2.4, 5.4	
500–999	6.1	4.3, 8.6	
≥1000	6.8	4.6, 10.0	
Demographic variables			
Sex			<0.001 *
Male	5.9	4.9, 7.0	
Female	9.2	7.6, 10.9	
Age (years)			<0.001 *
15–19	8.1	4.9, 12.9	
20–30	5.8	4.4, 7.7	
31–45	5.4	4.3, 6.9	
46–60	9.2	7.5, 11.1	
≥61	12.3	8.1, 18.2	
Education			0.494
Elementary	7.6	5.9, 9.8	
Secondary	6.5	5.3, 7.9	
Bachelor and beyond	7.5	6.1, 9.3	
Religion			0.872
Buddhism	7.1	6.2, 8.0	
Others	6.7	2.3, 17.8	
Monthly income (THB)			0.045 *
<5000	9.8	7.1, 13.3	
5000–9999	8.2	6.6, 10.1	
10,000–19,999	5.7	4.6, 7.2	
20,000–29,999	6.6	4.6, 9.3	
≥30,000	6.0	3.4, 10.4	

* *p*-value < 0.05; ^t^ Chi-squared.

## Data Availability

Dataset is available on request from the authors with an approval of access by the Center for Alcohol Studies, Thailand (required).

## Supplementary Materials

The following supporting information can be downloaded at https://www.mdpi.com/article/10.3390/ijerph23070912/s1, Table S1. Demographic characteristics of the sample by survey waves. Table S2. Factors associated with intention to quit drinking excluding completing former-year campaign as a covariate. Table S3. Sensitivity analysis 1: factors associated with intention to quit drinking (all covariates). Table S4. Sensitivity analysis 2: factors associated with intention to quit drinking (all covariates + year of survey).

## Abbreviations

The following abbreviations are used in this manuscript:

95% CI	95% confidence interval
AIC	Akaike Information Criterion
AOR	Adjusted odds ratio
AUDIT-C	Alcohol Use Disorders Identification Test–Concise
CAS	Center for Alcohol Studies
OR	Odds ratio
THB	Thai baht
UK	United Kingdom
WHO	World Health Organization
