# Supplementary material for: Beyond Temporary Sobriety: The Association Between Sobriety Campaign Completion and Intention to Quit Alcohol
Source: Int J Environ Res Public Health. 2026 Jul 16;23(7):912. doi: 10.3390/ijerph23070912 (PMC13410181; doi:10.3390/ijerph23070912)
Supplement: Supplementary file 1 [file ijerph-23-00912-s001.zip › ijerph-4327486-supplementary.pdf]

Supplementary

Table S1. Demographic characteristics of the sample by survey waves

Characteristic	Total		2015		2018		2021	
	**n**	**%**	**n**	**%**	**n**	**%**	**n**	**%**
**Total**	**3283**	**100.0**	**1883**	**100.0**	**924**	**100.0**	**476**	**100.0**
**Demographic variables**								
**Sex**								
Male	2093	63.8	1,224	65.0	610	66.0	259	54.4
Female	1190	36.2	659	35.0	314	34.0	217	45.6
**Age (years)**								
15-19	189	5.8	137	7.3	32	3.5	20	4.2
20-30	839	25.6	508	27.0	212	23.0	119	25.0
31-45	1184	36.1	684	36.4	331	35.9	169	35.5

46-60	898	27.4	445	23.7	319	34.6	134	28.2
≥61	167	5.1	106	5.6	27	2.9	34	7.1
**Education**								
Elementary	751	23.0	539	28.8	115	12.4	97	20.5
Secondary	1470	45.0	755	40.4	476	51.6	239	50.4
Bachelor and beyond	1045	32.0	575	30.8	332	36.0	138	29.1
**Religion**								
Buddhism	3203	98.6	1,826	98.7	917	99.2	460	96.8
Others	46	1.4	24	1.3	7	0.8	15	3.2
**Monthly income (THB)**								
<5,000	367	11.3	284	15.3	45	4.9	38	8.0
5,000-9,999	985	30.3	647	34.8	210	22.8	128	26.9
10,000-19,999	1272	39.1	624	33.6	430	46.6	218	45.9

20,000-29,999	446	13.7	210	11.3	165	17.9	71	14.9
≥30,000	186	5.7	94	5.1	72	7.8	20	4.2
**<u>Campaign-related variables</u>**								
**Exposure to campaign media**								
No	407	12.4	125	6.6	169	18.3	113	23.7
Yes	2876	87.6	1,758	93.4	755	81.7	363	76.3
**Completing recent-year campaign**								
No	1718	52.3	1,026	54.5	419	45.3	273	57.4
Yes	1565	47.7	857	45.5	505	54.7	203	42.6
**Completing former-year campaign**								
No	1847	56.4	1,070	57.1	490	53.0	287	60.6

Yes	1426	43.6	805	42.9	434	47.0	187	39.5
**<u>Drinking-related variables</u>**								
**Intention to quit drinking**								
No	2984	92.9	1,711	92.5	834	92.2	439	96.1
Yes	227	7.1	138	7.5	71	7.8	18	3.9
**Drinking expenses per occasion (THB)**								
<300	1470	50.1	718	45.6	443	49.2	309	67.0
300-499	623	21.2	295	18.7	237	26.3	91	19.7
500-999	502	17.1	309	19.6	147	16.3	46	10.0
≥1000	341	11.6	252	16.0	74	8.2	15	3.3
**Drinking frequency prior to the campaign**								

Weekly	998	30.6	442	23.8	387	41.9	169	35.7
Monthly	1275	39.1	805	43.3	284	30.8	186	39.2
Occasionally	985	30.2	614	33.0	252	27.3	119	25.1
**Past-year episode of drunkenness**								
Never	1371	41.9	840	44.8	343	37.2	188	39.7
Occasionally	1167	35.6	656	34.9	361	39.1	150	31.6
3+ times	736	22.5	381	20.3	219	23.7	136	28.7

Table S2. Factors associated with intention to quit drinking excluding completing former-year campaign as a covariate

Variable	AOR	95%CI	p-value	LR test
Campaign-related variables				
Completing recent-year campaign				<0.001*
No	1			
Yes	4.75	3.17, 7.34	<0.001*	
Exposure to campaign media				0.899
No	1			
Yes	0.97	0.61, 1.62	0.899	
Drinking-related variables				
Drinking frequency				<0.001*
Weekly	1			
Monthly	1.19	0.71, 2.03	0.519	

Occasionally	3.57	2.17, 6.03	<0.001*	
Past-year episode of drunkenness				0.483
Never	1			
Occasionally	0.81	0.56, 1.15	0.242	
≥3 times	0.85	0.48, 1.44	0.548	
Drinking expenses per occasion (THB)				0.089
<300	1			
300-499	0.57	0.34, 0.92	0.026*	
500-999	0.84	0.53, 1.30	0.449	
≥1000	1.13	0.66, 1.87	0.651	
Demographic variables				
Sex				

Male	1			
Female	1.01	0.72, 1.41	0.949	0.949
Age (years)				<0.001*
15-19	1			
20-30	1.01	0.47, 2.36	0.974	
31-45	1.18	0.54, 2.80	0.690	
46-60	2.31	1.06, 5.45	0.043*	
≥61	3.00	1.22, 7.69	0.019*	
Education				0.164
Elementary	1			
Secondary	0.97	0.64, 1.46	0.866	
Bachelor and beyond	1.37	0.89, 2.13	0.161	
Religion				0.649
Buddhism	1			

Others	0.71	0.11, 2.64	0.661	
Monthly income (THB)				0.013*
<5,000	1			
5,000-9,999	0.76	0.45, 1.32	0.316	
10,000-19,999	0.42	0.24, 0.75	0.003*	
20,000-29,999	0.56	0.29, 1.10	0.090	
≥30,000	0.45	0.18, 1.03	0.067	
**AIC value = 1190.05**				

*p-value <0.05

Note. n = 2820 (463 records contained missing data); AOR = Adjusted odds ratio; LR test = Likelihood ratio test; AIC = Akaike Information Criterion.

Table S3. Sensitivity analysis 1: factors associated with intention to quit drinking (all covariates)

Variable	AOR	95%CI	p-value	LR test
Campaign-related variables				
Completing recent-year campaign				<0.001*
No	1			
Yes	5.81	3.45, 9.92	<0.001*	
Completing former-year campaign				0.222
No	1			
Yes	0.75	0.48, 1.19	0.217	
Exposure to campaign media				0.887
No	1			
Yes	0.97	0.60, 1.61	0.887	
Drinking-related variables				

Drinking expenses per occasion (THB)				0.090
<300	1			
300-499	0.56	0.33, 0.91	0.025*	
500-999	0.83	0.52, 1.28	0.407	
≥1000	1.10	0.64, 1.83	0.719	
Drinking frequency				<0.001*
Weekly	1			
Monthly	1.20	0.71, 2.06	0.490	
Occasionally	3.64	2.22, 6.16	<0.001*	
Past-year episode of drunkenness				0.456
Never	1			
Occasionally	0.80	0.56, 1.14	0.228	
3+ times	0.83	0.47, 1.42	0.512	

Demographic variables				
Sex				0.906
Male	1			
Female	1.02	0.73, 1.42	0.906	
Age (years)				<0.001*
15-19	1			
20-30	1.00	0.46, 2.33	0.997	
31-45	1.19	0.54, 2.81	0.682	
46-60	2.35	1.08, 5.55	0.039*	
≥61	3.09	1.25, 7.93	0.016*	
Education				0.184
Elementary	1			
Secondary	0.96	0.64, 1.46	0.862	
Bachelor and beyond	1.35	0.88, 2.11	0.177	

Religion				0.607
Buddhism	1			
Others	0.68	0.10, 2.55	0.623	
Monthly income (THB)				0.015*
<5,000	1			
5,000-9,999	0.77	0.45, 1.35	0.356	
10,000-19,999	0.43	0.24, 0.77	0.004*	
20,000-29,999	0.58	0.30, 1.14	0.110	
≥30,000	0.46	0.19, 1.05	0.073	
**AIC value = 1190.35**				

*p-value <0.05

Note. n = 2817 (466 records contained missing data); AOR = Adjusted odds ratio; LR test = Likelihood ratio test; AIC = Akaike Information Criterion.

Table S4. Sensitivity analysis 2: factors associated with intention to quit drinking (all covariates + Year of survey)

Variable	AOR	95%CI	p-value	LR test
Campaign-related variables				
Completing recent-year campaign				<0.001*
No	1			
Yes	5.45	3.23, 9.31	<0.001*	
Completing former-year campaign				0.260
No	1			
Yes	0.77	0.50, 1.22	0.255	
Exposure to campaign media				0.835
No	1			
Yes	0.95	0.59, 1.60	0.834	
Drinking-related variables				

Drinking expenses per occasion (THB)				0.070
<300	1			
300-499	0.54	0.32, 0.88	0.017*	
500-999	0.81	0.51, 1.26	0.355	
≥1000	1.06	0.61, 1.76	0.840	
Drinking frequency				<0.001*
Weekly	1			
Monthly	1.28	0.75, 2.19	0.371	
Occasionally	3.71	2.25, 6.29	<0.001*	
Past-year episode of drunkenness				0.345
Never	1			
Occasionally	0.77	0.53, 1.10	0.151	
3+ times	0.84	0.47, 1.43	0.531	

Demographic variables				
Sex				0.821
Male	1			
Female	1.04	0.74, 1.45	0.821	
Age (years)				<0.001*
15-19	1			
20-30	0.98	0.45, 2.29	0.964	
31-45	1.16	0.53, 2.75	0.721	
46-60	2.27	1.04, 5.35	0.049*	
≥61	3.10	1.26, 7.99	0.015*	
Education				0.170
Elementary	1			
Secondary	0.93	0.62, 1.41	0.716	
Bachelor and beyond	1.32	0.85, 2.07	0.214	

Religion				0.720
Buddhism	1			
Others	0.76	0.11, 2.90	0.728	
Monthly income (THB)				0.013*
<5,000	1			
5,000-9,999	0.77	0.45, 1.35	0.354	
10,000-19,999	0.42	0.24, 0.76	0.003*	
20,000-29,999	0.57	0.29, 1.13	0.107	
≥30,000	0.44	0.18, 1.02	0.062	
Year of survey				0.055
2015	1			
2018	1.31	0.91, 1.88	0.147	
2021	0.63	0.34, 1.10	0.122	

**AIC value = 1190.67**				

*p-value <0.05

Note. n = 2817 (466 records contained missing data); AOR = Adjusted odds ratio; LR test = Likelihood ratio test; AIC = Akaike Information Criterion.
